# Textures on the surface of BSA films with different concentrations of sodium halides and water state in solution

**DOI:** 10.1186/s11671-015-0860-0

**Published:** 2015-03-28

**Authors:** Gennadiy Glibitskiy, Dmitriy Glibitskiy, Olga Gorobchenko, Oleg Nikolov, Alexander Roshal, Mikhail Semenov, Anatoliy Gasan

**Affiliations:** A.Ya. Usikov Institute for Radiophysics and Electronics, National Academy of Sciences of Ukraine, 12, Academician Proskura Str, Kharkiv, 61085 Ukraine; Department of Molecular and Medical Biophysics, School of Radiophysics, V.N. Karazin Kharkiv National University, 4 Svobody Sq, Kharkiv, 61022 Ukraine; Institute for Chemistry, V.N. Karazin Kharkiv National University, 4 Svobody Sq, Kharkiv, 61022 Ukraine

**Keywords:** Biopolymers, Films, BSA, Textures, Microwave dielectrometry, Hydration

## Abstract

The formation of the textures on the surface of the films from the solutions of bovine serum albumin (BSA) with sodium halides (NaF, NaCl, and NaBr) of various concentrations was studied. The formation of symmetric zigzag textures on the surface of BSA films (Cryst Eng 3:173-194, 2000) in the presence of sodium halides depends on the conformational state of the protein globule. Thermal denaturation of BSA also did not allow to form zigzag textures on the surface of the films.

## Background

The structure of the solid phase of a drying biopolymer fluid characterizes the interaction between the substances in the solution [1,2]. On the surface of the films obtained from the solutions of biopolymers, fractal textures are formed; it is assumed that such structures appear due to the formation of complex crystalline hydrate of proteins and salts [3,4]. Theoretical models of the processes leading to the formation of such textures are presented in [5,6].

It is assumed that these structures are formed in the process of self-organization of DNA on the film surface, when the biopolymer is in the appropriate conformational state and the corresponding hydration environment.

The area of textures decreases as the result of DNA denaturation caused by the presence of silver ions in the solution [7].

In [8], it is shown that chlorine ions promote the condensation of sodium ions around the DNA, and Na^+^ permeates into the DNA double helix, which facilitates the assembly of DNA molecules into compact structures.

The area of textures on a film decreases [9] with the decrease of NaCl concentration in the source solution.

It is shown that zigzag (Z) structures are formed on films produced from solutions of DNA with NaCl at relative humidity (RH) levels of 11% to 45% [10].

Solutions containing bovine serum albumin (BSA) and potassium chloride result in similar structures under such conditions [11]. These conditions are likely to be closely related to the nature of electrostatic interactions in the water-biopolymer-ions system, as these interactions cause the formation of supramolecular structures (dynamic biopolymer complexes) in a certain range of concentrations of biopolymer and salt [12]. The ability to create such complexes may underlie the formation of a particular type of textures on the films during the water-biopolymer-ions system’s desiccation. The change in the nature of electrostatic interactions in such systems (for example, as a result of adding heavy metal ions, even in small amounts) hinders the formation of Z-structures on films from the solutions of BSA and NaCl [13].

In [14], it is shown that, in lysozyme solution with NaCl, protein aggregates are formed at the stage of evaporation.

In [15], it is shown that cations and anions are bound to the surface of BSA globule. The calculation of surface charge that promotes the formation of a layer of counterions is presented in [16]. Structure formation in drying drops of saline solutions of protein is discussed in the paper [17]. There, it was shown that these complex structures are formed from salt.

In the paper [18], the conclusion was made that the adhesion of protein molecules to the surface of a nascent crystal leads to the formation of dendrite.

On the other hand, [19,20] describe a thermodynamic instability in the protein solution at salt concentrations above physiological, which leads to the formation of various unstable supramolecular structures, such as protein fractal clusters and the nuclei of the crystalline phase.

The general patterns of interaction between water, ions and biomacromolecules are not yet fully understood; the role of the state of water, which can adjust the balance in such systems, has not been established. Also, the role of conformation of the biopolymer during the formation of fractal textures on the surface of desiccating films is not yet clarified.

Therefore, the aim of this work is to study the formation of textures on the surface of films from solutions containing BSA and sodium halide salts (NaF, NaCl, and NaBr), as well as to study the state of water in the water-BSA-salt system and the change in the BSA conformation. Microwave dielectrometry was used to study the structure of water [21], and the conformational state of the BSA was studied by UV and fluorescence spectroscopy.

## Methods

### Preparation of aqueous solutions of BSA containing sodium halides

In the experiment, BSA preparations (DiaM, USA) and sodium halide salts (NaF, NaCl, and NaBr, xv grade) were used. Aqueous solutions of the protein and salts were prepared in distilled water.

For the production of films, solutions of BSA (at a concentration of 0.5 mg/ml) containing sodium halide salts (NaF, NaCl, and NaBr) at a concentration of 20 mM were used.

For microwave dielectrometry measurements, as well as UV and fluorescence spectroscopy, samples were prepared with a greater concentration of BSA (10 mg/ml) and salt (0.4 M); the BSA/salt ratio remained the same as in the samples from which the films were obtained. The solutions were prepared as follows. Initially, the source solutions of 20 mg/ml of BSA and 0.8 M of each salt were prepared. For this, the required amount of distilled water was added to the corresponding weigh of salt or BSA. After BSA and salts had dissolved, the solutions were mixed in the volume ratio of 1:1; then, the samples were stirred continuously for 2 h with a magnetic stirrer at a room temperature (about 20°C). The control samples of BSA and salt solutions were prepared by mixing the source solutions of BSA and salts with distilled water in the 1:1 ratio.

To investigate the effect of BSA conformation on the formation of textures, a denaturation of BSA solution with NaCl was carried out at 70°C and 95°C for 10 and 15 min, respectively.

The fluorescence emission intensity of BSA at *λ* = 345 nm is known to be quenched as a result of thermal denaturation [22] and gamma irradiation [23]. The fluorescence spectra of the samples we studied exhibit a similar behavior, with the 70°C and 95°C heated solutions having 28% to 35% lower fluorescence intensity than non-heated ones.

### Preparation of films

The formation of textures on BSA films was performed using the setup described in [7,9]. Of the corresponding solutions, 0.5 ml is poured into cells made of quartz glass. The area of a cell is 20 × 21 mm^2^, and the height of the cell walls is 1 mm. The cell is placed in a sealed container which has entries for pumping air with a specific relative humidity (RH), as well as temperature and humidity sensors. The container is placed in a water bath. The accuracy of temperature stabilization is 0.5°C, and the accuracy of moisture determination is 2% to 3%.

### Analysis of the distribution of textures on films

This paper considers the numerical criterion that is based on the recognition of a specific type of texture, and analyzes the nature of texture change when the concentration of ions changes. The specific length of zigzags $\overline{L}$ is proposed as a numerical characteristic of the abovementioned textures. The calculation of this parameter was carried out as follows: 1) *n* micrographs were taken at positions that are uniformly distributed over the area of the cell; 2) for each photo, the zigzag texture elements were manually marked; and 3) the final value of $\overline{L}$ was determined as:

$$ \overline{L}=\frac{{\displaystyle {\sum}_i^n}\frac{L_i}{S_i}}{n}, $$

where *L*_*i*_ is the total length of zigzags in a single photograph, *S*_*i*_ is the spot area of the microscope for the photo, and *n* is the number of photos.

For taking the micrographs, a Meopta microscope (Meopta - optika, s. r. o., Prerov, Czech Republic) with Logicfox LF-PC011 web-camera and custom capturing software were used.

### UV and fluorescence spectroscopy

UV absorption spectra of the BSA solutions containing sodium halide salts were obtained in the 250- to 300-nm wavelength range using a UV spectrophotometer Hitachi U2310 (Hitachi, Ltd, Chiyoda-ku, Japan). The fluorescence spectra of the water-BSA-salt solutions and the films corresponding to these solutions were obtained in the 290- to 460-nm wavelength range using a spectrofluorometer Hitachi 850 (Hitachi, Ltd, Chiyoda-ku, Japan). The relative fluorescence intensities of the samples were approximately evaluated via comparing the values obtained by dividing the fluorescence intensity at the maximum of BSA emission band by the optical density at the maximum of BSA absorption band at *λ* = 280 nm.

### Microwave dielectrometry

The real *ε′* and imaginary *ε″* parts of complex permittivity of the BSA solution (10 mg/ml), solutions of sodium halides NaF, NaCl, and NaBr (0.4 M), and water-BSA-salt systems (10 mg/ml BSA and 0.4 M salt) were measured using the microwave dielectric method [21] at a frequency of 9.2 GHz and a temperature of 22.9°C ± 0.1°C. Corrections for the presence of inorganic ions were made on the basis of conductivity measurements at a frequency of 1 kHz. The dielectric relaxation frequency *f*_*d*_ (*f*_*d*_ = 1/(2*πτ*), where *τ* is the relaxation time) of water molecules in solutions, and the static dielectric constant *ε*_*s*_ (permittivity of the solution in the low-frequency region of water relaxation) were calculated from the obtained *ε′* and *ε″* values using the expressions derived from the Debye equation [24]:

$$ {f}_d=\frac{f\left({\varepsilon}^{\hbox{'}}-{\varepsilon}_{\infty}\right)}{\varepsilon^{\hbox{'}\hbox{'}}}, $$

$$ {\varepsilon}_s={\varepsilon}^{\hbox{'}}+{\varepsilon^{\hbox{'}\hbox{'}}}^2/\left({\varepsilon}^{\hbox{'}}-{\varepsilon}_{\infty}\right), $$

where *f* is the frequency of the microwave field and *ε*_*∞*_ = 5.6 is water permittivity in the infrared frequency range [25].

## Results and discussion

**Figure 1:**
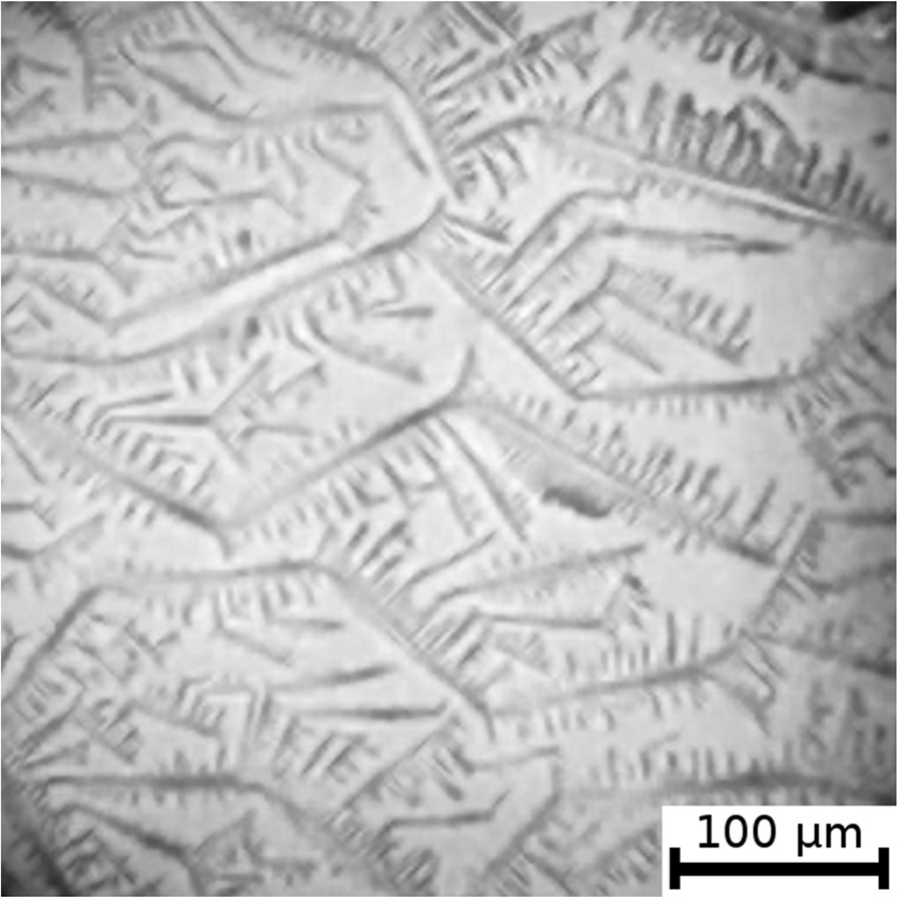
**Film from BSA + 20 mM NaCl solution; T = 40°C, RH = 0%, central part.**

**Figure 2:**
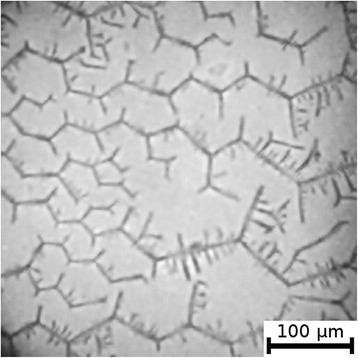
**Film from BSA + 20 mM NaCl solution; T = 40°C, RH = 0%, peripheral part.**

**Figure 3:**
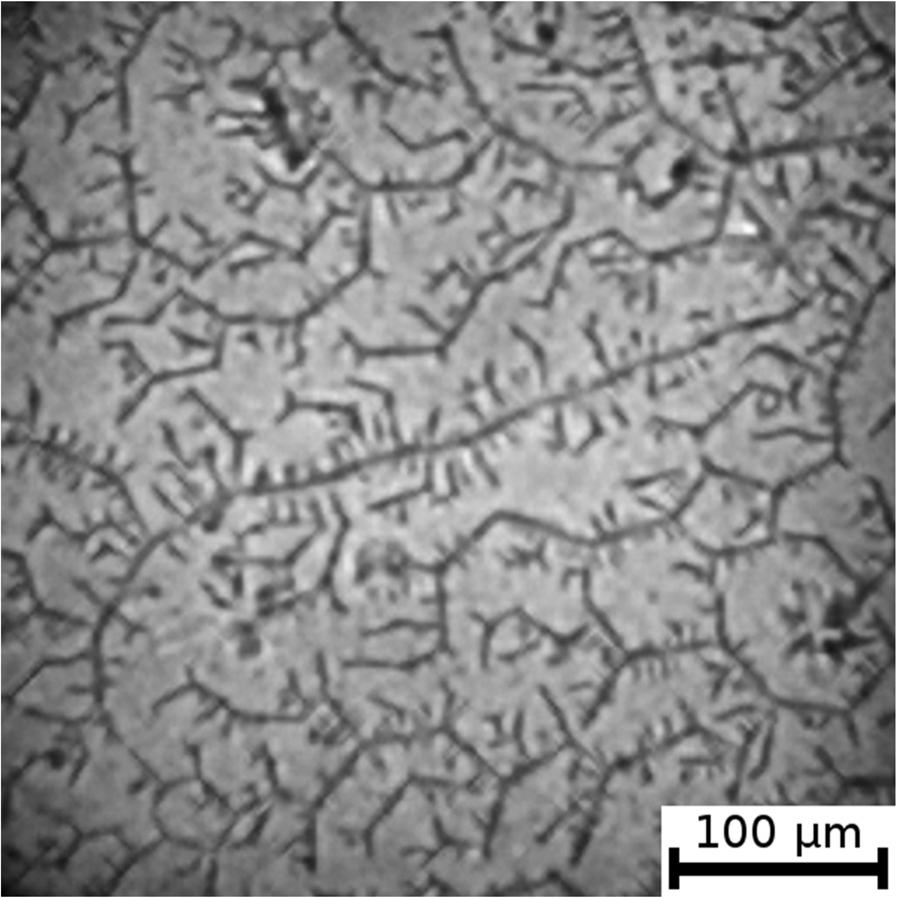
**Film from BSA + 12 mM NaCl + 8 mM NaF solution; T = 40°C, RH = 0%.**

**Figure 4:**
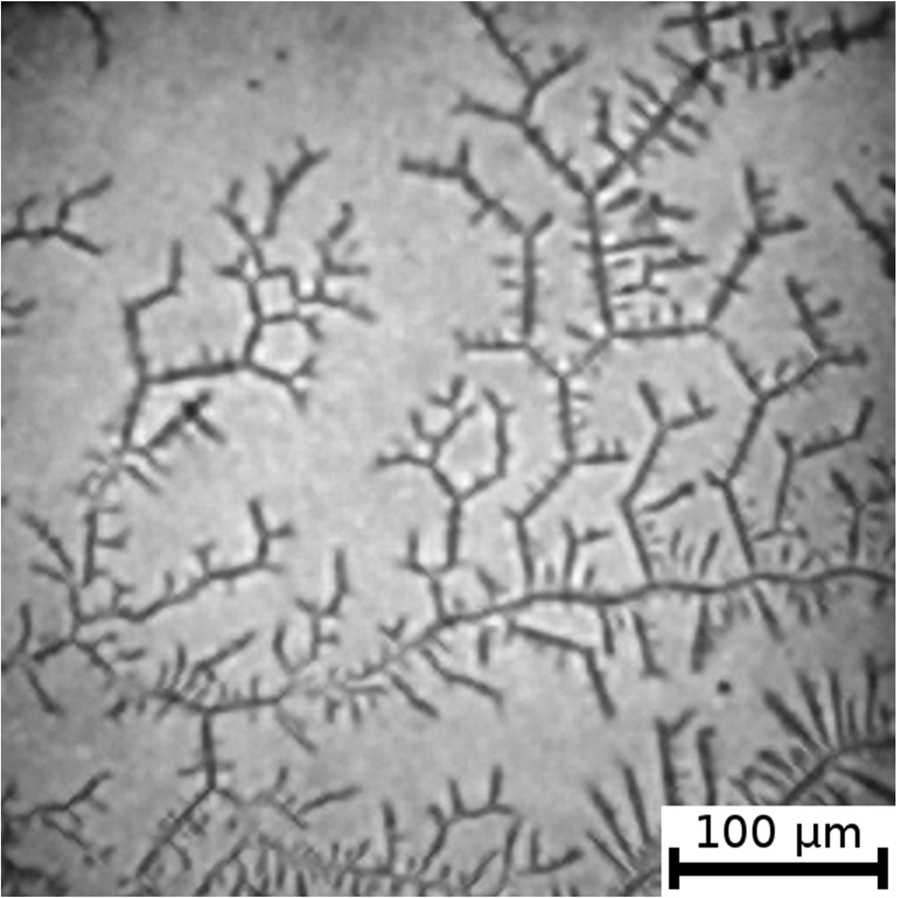
**Film from BSA + 12 mM NaCl + 8 mM NaBr solution; T = 40°C, RH = 0%.**

**Figure 5:**
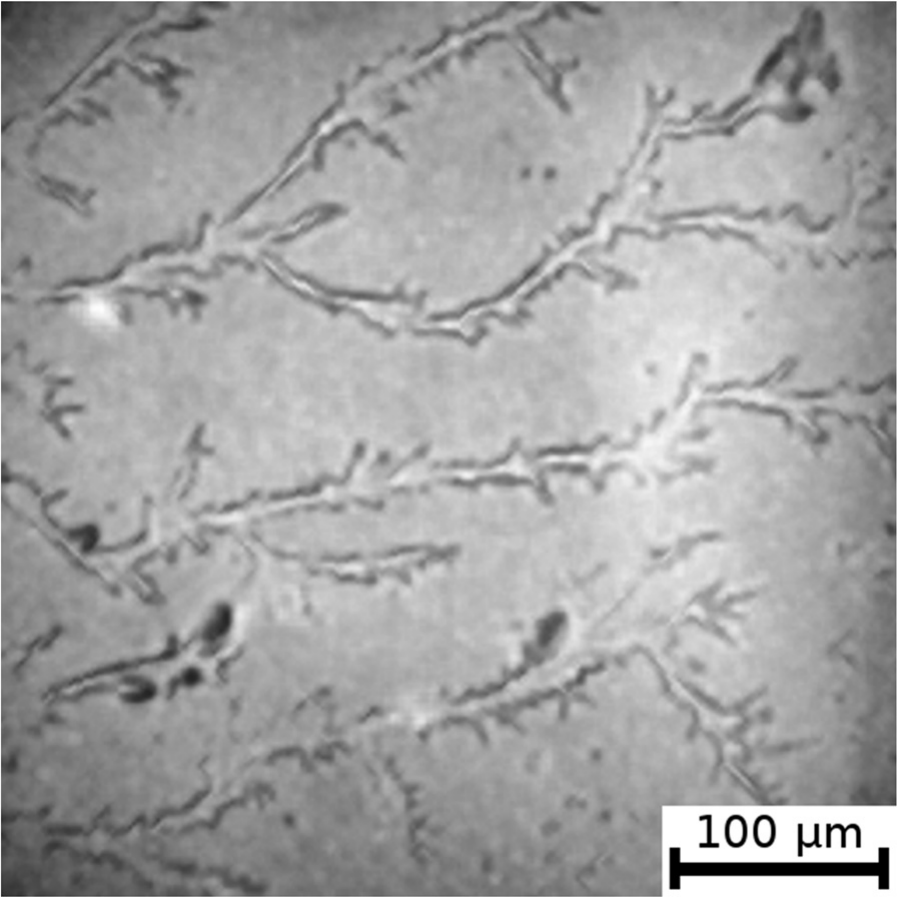
**Film from BSA + 20 mM NaF solution; T = 40°C, RH = 0%.**

**Figure 6:**
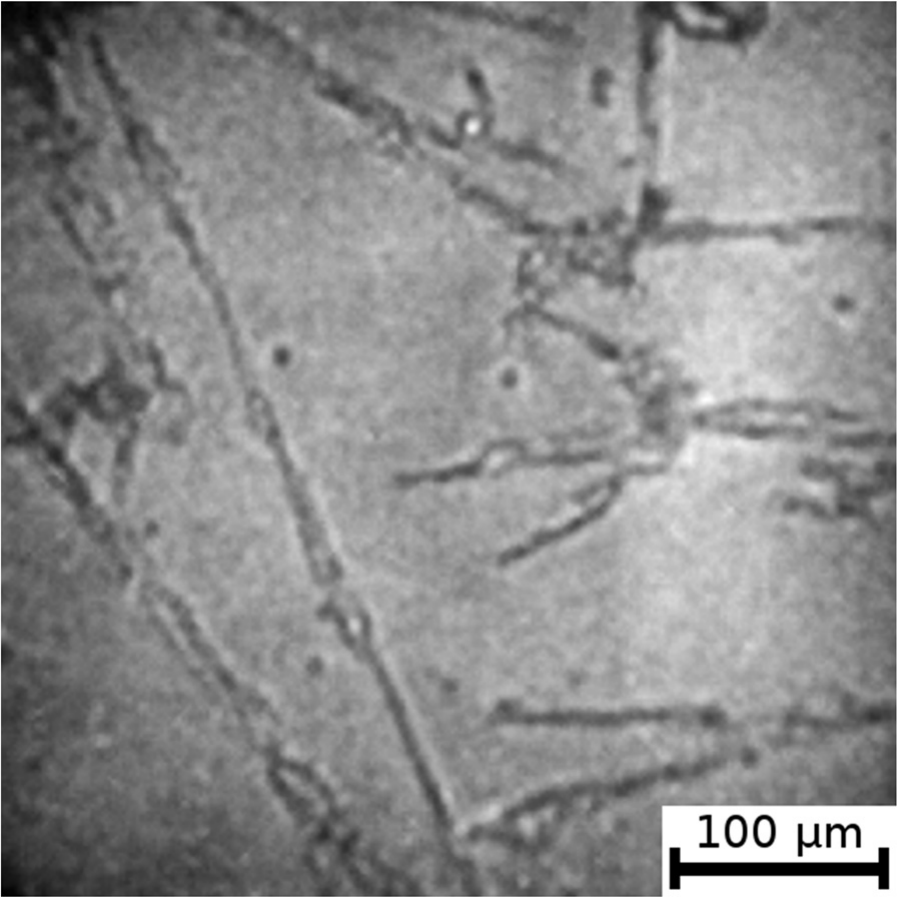
**Film from BSA + 20 mM NaBr solution; T = 40°C, RH = 0%.**

**Figure 7:**
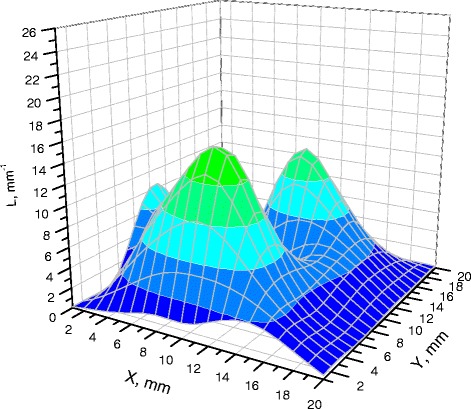
**Distribution of densities of Z-structures in BSA films (12 мМ NaCl; 8 мМ NaBr).**

**Figure 8:**
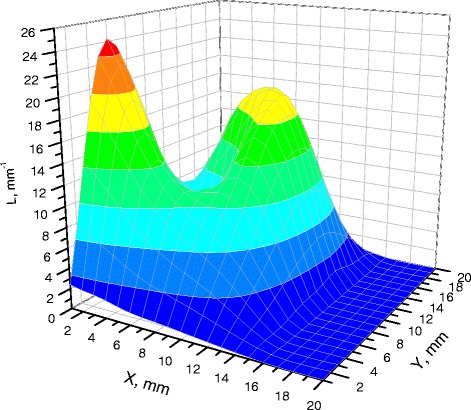
**Distribution of densities of Z-structures in BSA films (12 мМ NaCl; 8 мМ NaF).**

The textures of the films of BSA solutions with NaF, NaCl and NaBr are shown in Figures 1, 2, 3, 4, 5, and 6. The distribution of Z-structures and the value of specific length $\overline{L}$ varied with the concentration of Cl^−^ in the source solutions decreasing from 20 to 0 mM and the concentration of fluorine and bromine ions increasing from 0 to 20 mM. The distribution of textures on the films’ surface is shown in Figures 7 and 8 in the form of three-dimensional graphs.

**Figure 9:**
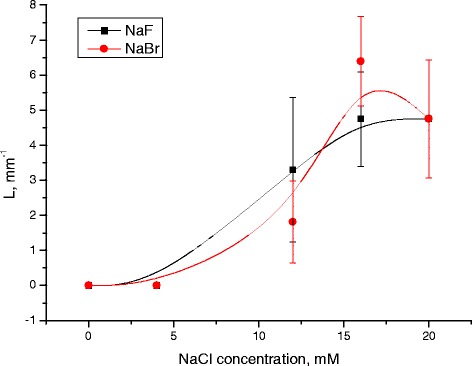
**Dependency of specific length of zigzags on Cl** ^**−**^ **concentration.**

As can be seen, the number of Z-structures decreases when the concentration of Cl^−^ decreases, and F^−^ or Br^−^ is present in the starting solution (Figures 1, 2, 3, 4, 5, 6, 7, and 8). The calculations of Z-structures’ density distribution (Figure 9) suggest that a decrease in the specific length of zigzags $\overline{L}$ correlates with a decrease in the concentration of Cl^−^ in the solution. From this, we can conclude that it is the presence of Cl^−^ in the solution that creates conditions under which the fractal structures are formed during the films’ desiccation. On the other hand, the presence of F^−^ or Br^−^ in the solution reduces the amount of Z-structures in the film.

**Figure 10:**
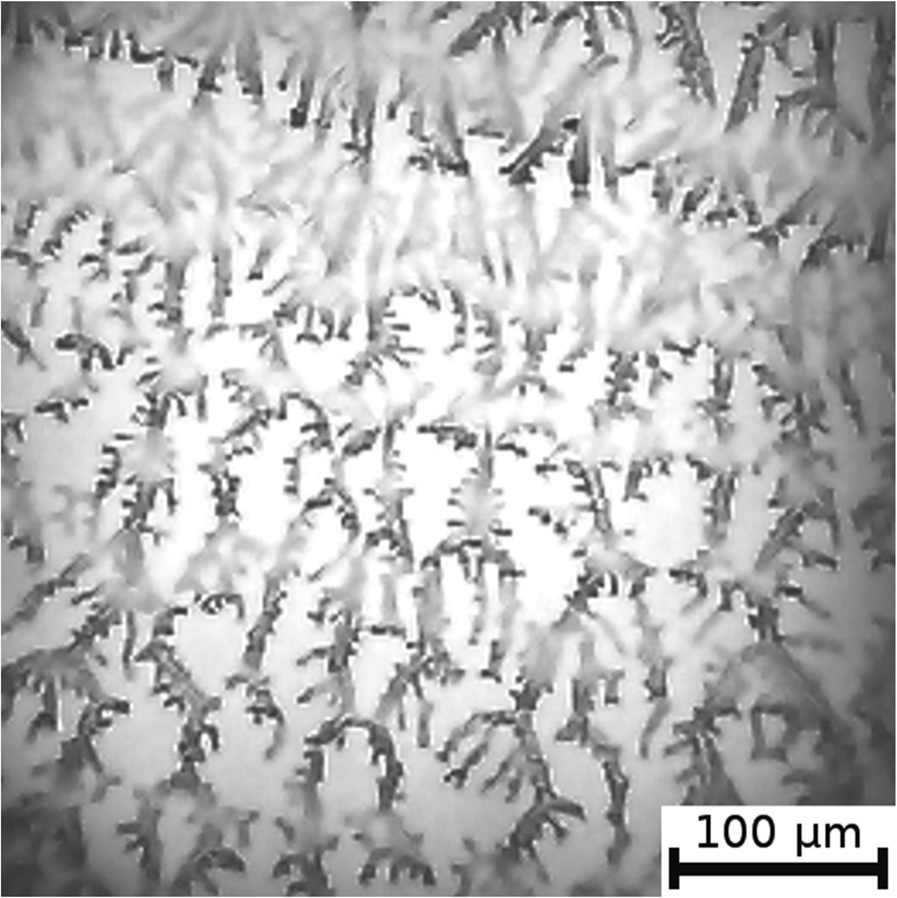
**Photo of the film obtained from solution containing BSA + 20 mM NaCl;** ***T*** **= 70°C.**

**Figure 11:**
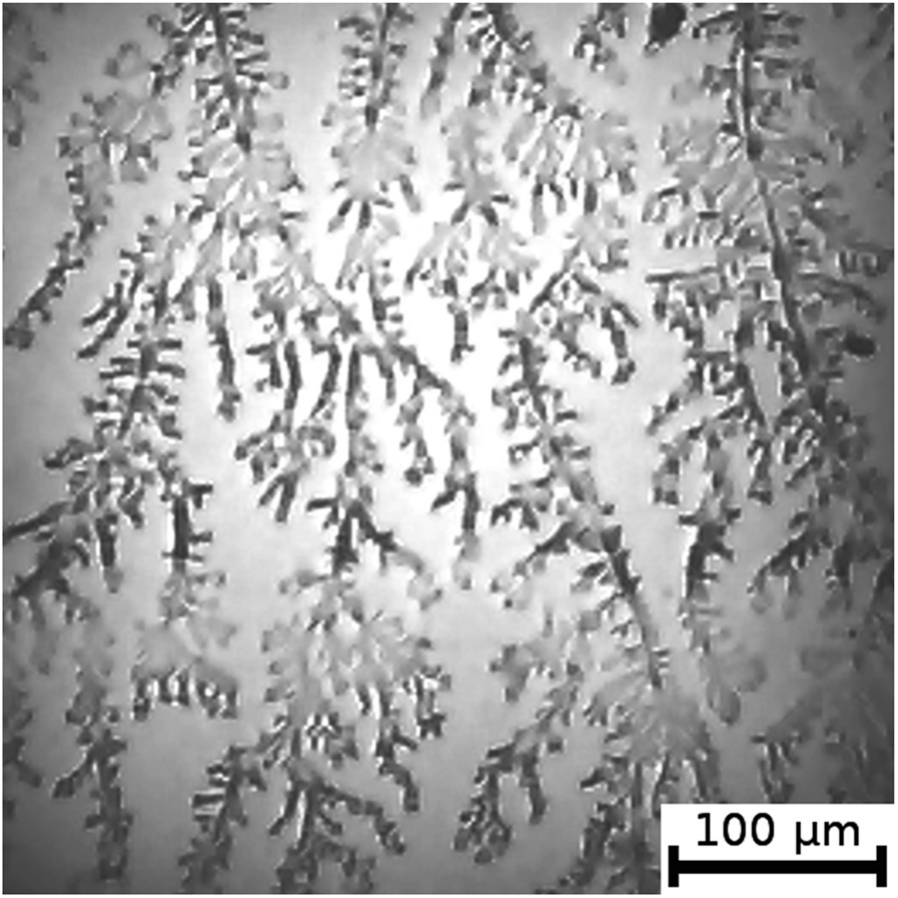
**Photo of the film obtained from solution containing BSA + 20 mM NaCl;** ***T*** **= 95°C.**

The photos of the films obtained from solutions containing BSA + 20 mM NaCl at *T* = 70°C and *T* = 95°C are provided in Figures 10 and 11. The value of $\overline{L}$ for 70°C is 2.26 ± 0.40 mm^−1^, and for 95°C, it is 0 mm^−1^.

**Figure 12:**
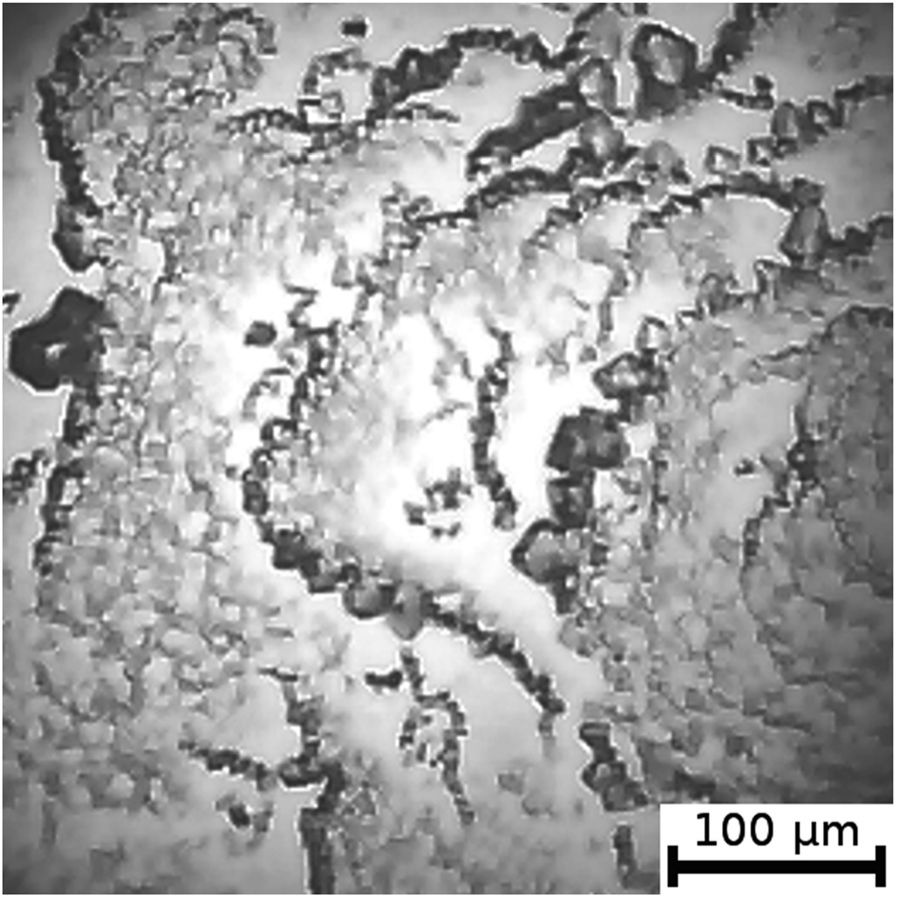
**Photo of the film obtained from solution containing 20 mM NaCl.**

As can be seen from Figure 12, Z-structures are not formed in the case of salt solutions without BSA. Z-structures also do not form in the case of solution containing denatured BSA.

It is known that interactions between biopolymers and salts cause the displacement of water molecules from the hydration shell of protein molecules, as well as the alteration of the spatial conformation of the resultant structure. Certain salts destabilize and others stabilize the structure of biopolymers and influence their degree of hydration [26]. The difference in halides’ effect on BSA is, possibly, due to the changes in the degree of hydration and the conformation of BSA, as well as the nature of electrostatic interactions in the BSA-ion [27]. Indeed, Figures 5 and 6 show that there are no fractal structures on the surface of BSA films in the presence of F^−^ and Br^−^.

**Figure 13:**
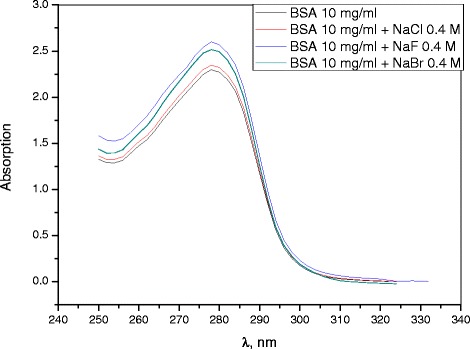
**Absorption spectra.**

To clarify the nature of influence of F^−^, Cl^−^, and Br^−^ on the conformation of BSA globules, we have conducted a study of UV spectra and fluorescence spectra of BSA solutions in the presence of sodium halide salts (NaF, NaCl, and NaBr). Figure 13 shows the absorption spectra of BSA near the *λ* ~ 280 nm absorption band, which is known to be sensitive to the conformational state of protein. It can be seen that, in the presence of F^−^ and Br^−^, the absorption band intensity increases by about 8% (±2%). This suggests that these ions modify not just the surface environment of a protein globule, but also its conformation.

**Figure 14:**
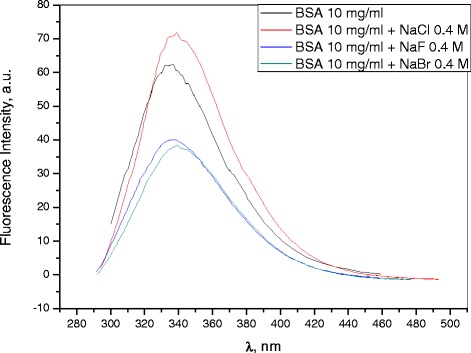
**Fluorescence intensity spectra.**

Figure 14 shows the fluorescence spectra of BSA solutions in the presence of sodium halides. As was mentioned above, the samples’ emission bands were compared by their ‘effective’ fluorescence intensity: the ratio ${I}^{\hbox{'}}=\frac{I_{\max}^{\mathrm{fl}}}{A_{\mathrm{exc}}}$ of intensity ${I}_{\max}^{\mathrm{fl}}$ at the band’s emission maximum to the optical density *A*_*exc*_ at the point of excitation (280 nm). Table 1 shows that, in the presence of NaCl, *I*′ increases, whereas in the presence of NaF and NaBr, *I*′ decreases (by a similar amount in both cases). It is known that the luminescence of BSA is primarily due to tryptophan (BSA molecule contains two tryptophan residues that contribute significantly to the protein’s fluorescence: Trp-134, which is localized near the surface of the IB domain and Trp-212, which is immersed in a hydrophobic pocket in the inner part of the IIA domain [28]). The fluorescence intensity may decrease (fluorescence quenching) or increase as a result of BSA interaction with ions. For instance, negative charges lower the fluorescence intensity, while positive charges have the opposite effect [29,30]. Only Trp-214 may be quenched by iodide, whereas Trp-134 is normally protected [31]. In addition, increasing the ionic strength of the solution with 2 M of NaCl reduced the quenching effect of iodide; this is explained as the result of reduced electrostatic attraction and, possibly, of the F^−^ and Br^−^ competition for the same binding sites in BSA [32]. Thus, the change in fluorescence intensity indicates the changes in the microenvironment around the chromophore molecule. In our case, a decrease in fluorescence intensity in the presence of NaF and NaBr salts can, apparently, be attributed to the same effects as iodide quenching - i.e., the fluorescence quenching by F^−^ and Br^−^ of Trp-134, which is located on the protein’s surface.

**Table 1:** **The ‘effective’ fluorescence intensity of the samples**

**Sample**	${I}^{\hbox{'}}=\frac{I_{\mathbf{max}}^{\mathbf{fl}}}{{\boldsymbol{A}}_{\mathbf{exc}}}$
BSA 10 mg/ml	0.33
BSA 10 mg/ml + NaBr 0.4 M	0.25
BSA 10 mg/ml + NaCl 0.4 M	0.44
BSA 10 mg/ml + NaF 0.4 M	0.25

**Figure 15:**
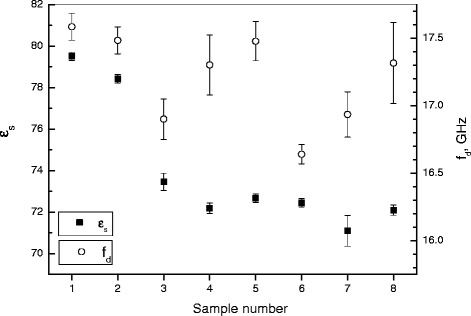
**The static dielectric permittivity** ***ε***_***s***_ **of the samples and the standard deviation (T = 22.9°C ± 0.1°C).** 1 - H2O; 2 - 10 mg/ml BSA soluted in H2O; 3 - 0.4 M NaF; 4 - 0.4 M NaCl; 5 - 0.4 M NaBr; 6 - 10 mg/ml BSA soluted in 0.4 M NaF; 7 - 10 mg/ml BSA soluted in 0.4 M NaCl; 8 - 10 mg/ml BSA soluted in 0.4 M NaBr.

We have investigated the state of water molecules surrounding BSA using microwave dielectrometry, which is known to be sensitive to water molecules in solution. Figure 15 shows the values of the test samples’ static dielectric constant *ε*_*s*_ and the dielectric relaxation frequency *f*_*d*_ of water molecules in them. In protein or salt solutions, *ε*_*s*_ is determined by the amount of free water. *f*_*d*_ characterizes the mobility of water molecules in the solution, which, in turn, is determined by the nature of intermolecular interactions. By considering both factors, we can obtain information about the state of the protein’s hydration shell in the samples. For instance, a 0.4 M solution of NaF has the maximum value of *ε*_*s*_ and the minimum value of *f*_*d*_ compared to other salt solutions. From this, we can conclude that the hydration number of F^−^ is less than the hydration numbers of Cl^−^ and Br^−^. The calculations of minimal hydration numbers by the method described in [33] have shown that NaF binds 10.5 water molecules, NaCl - 12.6, NaBr - 11.8. According to the method of molecular dynamics, the sum of average hydration numbers of the first hydration shell is 12.3 for Na^+^ and F^−^, 13.3 for Na^+^ and Cl^−^, and 13.1 for Na^+^ and Br^−^ [34].

Thus, the hydration numbers we obtained are below the average given by molecular dynamics. In our case, these are the water molecules that do not participate in the process of dipole relaxation. Their lifetimes are evidently above the average lifetimes (reported in [34]) of the water molecules in the ions’ first hydration shell. The dielectric relaxation frequency of the free water molecules in the salts’ solutions is less than in pure water.

This indicates that ions reduce the mobility of free water molecules in the solutions. In the F^−^, Cl^−^, Br^−^ series, the mobility of free water molecules increases, which is in agreement with the size and the surface charge of the ions. The difference in molecular mobility of free water is due to the different average number of H-bonds per water molecule at a given temperature.

Indeed, it is well known that Na^+^ and F^−^ increase and Cl^−^ and Br^−^ decrease the number of hydrogen bonds (in relation to pure water at the same temperature), with Br^−^ having a greater effect than Cl^−^ [35]. Our data is consistent with these facts. All this is also valid for solutions of BSA with NaF, NaCl, and NaBr.

In general, protein molecules and salts contribute additively to the *ε*_*s*_ and *f*_*d*_ values of BSA-salt solutions. It should also be noted that BSA solution with 0.4 M NaCl has the smallest value of *ε*_*s*_ compared to the other salts. This is associated with the greatest amount of bound water in this sample, which may play a key role in the formation of textures. After the evaporation of all the free water, the samples with different salts will contain different amounts of bound water. BSA molecules and ions likely compete for this water, with the nature of the competition depending on the amount of bound water in the system. This, in turn, will determine the nature of electrostatic interactions in the system and the nature of the interaction between its components. Ultimately, these interactions manifest at the macro level in the form of various types of textures growing on the surface of desiccating films.

After analyzing of the experiment results, it is impossible to reliably determine the mechanisms of interaction of BSA with salt during the evaporation process and the formation of textures.

However, it can be assumed that the formation of textures is in accordance with the model proposed by Tarasevich [18], wherein the growing salt crystal formation in the dendrite form is described as the result of adhesion of the protein molecules to the crystal surface.

In this case, various forms of dendrites (Z-form in particular) may appear due to the conformational differences of protein.

Since protein globule contains Na^+^ and Cl^−^ [15], we can also assume that fractal Z-textures appear due to the formation of protein-water-salt complex at a certain ratio of protein-salt, as demonstrated in [19,20].

## Conclusions

The study of the effect of different halides (NaF, NaCl, NaBr) on the formation of textures on the surface of BSA films has been conducted. It is shown that NaCl organizes the texture on the film surface by forming zigzag patterns, whereas NaF and NaBr hinder the formation of ordered Z-structures on the film. UV and fluorescence spectroscopies indicate that F^−^ and Br^−^ change the structural and the state of BSA globule, as opposed to Cl^−^. Thermal denaturation of protein prevented the appearance of symmetric (ordered) Z-structures on films. Thus, the formation of texture on the surface of BSA films in the presence of sodium halides depends on the conformational state of the protein globule. The question of the protein-salt or salt nature of the structure formation by NaCl crystals yet remains controversial.

## Abbreviations

BSA: bovine serum albumin
DNA: deoxyribonucleic acid
UV: ultraviolet
RH: relative humidity
